# Percutaneous Extraction of a Migrated WATCHMAN™ Device After Seven Months

**DOI:** 10.19102/icrm.2021.120701

**Published:** 2021-07-15

**Authors:** Abhishek Maan, Mohit K. Turagam, Srinivas R. Dukkipati, Vivek Y. Reddy

**Affiliations:** ^1^Helmsley Electrophysiology Center, Division of Cardiac Electrophysiology, Icahn School of Medicine at Mount Sinai, New York, NY, USA

**Keywords:** Atrial fibrillation, endoscopic grasping tool, device extraction, Watchman

## Abstract

Rarely, a left atrial appendage closure device may chronically migrate to an unfavorable position postoperatively, requiring removal. We present the details of a case in which a WATCHMAN™ device (Boston Scientific, Natick, MA, USA) implanted seven months prior was found to have migrated with protrusion 0.91 cm outside the left atrial appendage together with a 90° tilt and peridevice leakage. Adopting a femoral arterial retrograde approach, a 27-mm WATCHMAN™ device was temporarily positioned in the ascending aorta for cerebroembolic protection, never released from the connecting wire. Extraction of the original WATCHMAN™ device was performed using an endoscopic grasping tool, with subsequent device re-implantation of a new device and removal of the temporarily positioned device in the ascending aorta.

## Case presentation

**Figure 1:**
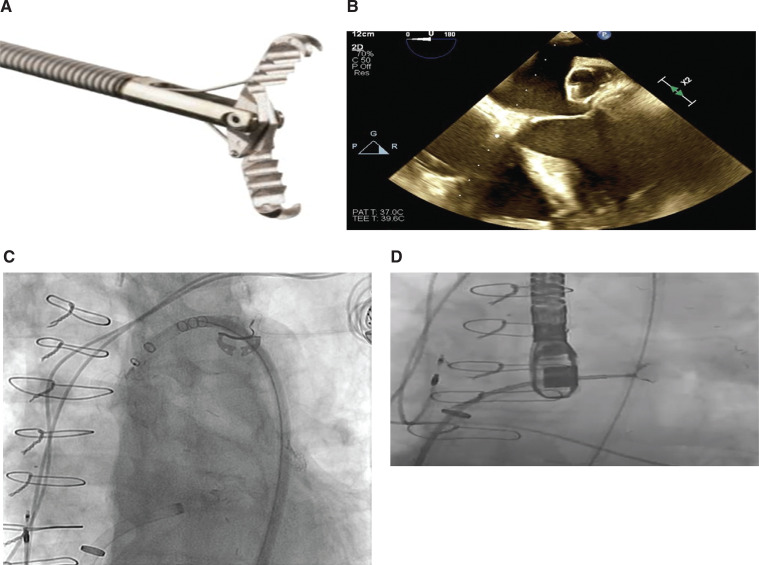
**A:** The endoscopic grasping tool with a hybrid jaw configuration combining both alligator and rat tooth capabilities for enhanced gripping. **B:** To remove the migrated WATCHMAN™ device, a second device was temporarily positioned in the ascending aorta for embolic protection **C:** and the grasping tool was used to retrieve the device. **D:** Grasping tool just prior to retrieving the WATCHMAN™ device.

An 84-year-old man with persistent atrial fibrillation (CHA_2_DS_2_-VASc score of six points) was referred because of the migration of a 24-mm WATCHMAN™ device (Boston Scientific, Natick, MA, USA) implanted seven months prior. His additional medical history was notable for chronic kidney disease and having undergone dual-chamber pacemaker implantation in 2017 for sinus node dysfunction. At the time of the current case, the patient was on a renally adjusted dose of rivaroxaban for thromboprophylaxis. Transesophageal echocardiography (TEE) revealed a loss of device compression with consequent protrusion 0.91 cm outside the left atrial appendage together with a 90º tilt and peridevice leakage **(Figure 1 and Videos 1A–1C)**.

Under general anesthesia, an extraction procedure was performed with TEE, intracardiac echocardiographic, and fluoroscopy guidance. Adopting a femoral arterial retrograde approach, a 27-mm WATCHMAN™ device was temporarily positioned in the ascending aorta for cerebroembolic protection, never released from the connecting wire (ie, “Watchman in Ascending Aorta for Systemic Protection” technique) **(Videos 2A and 2B)**.^1^ A weight-based heparin intravenous bolus dose was given to achieve an activated clotting time of greater than 250 seconds prior to pursuing transseptal access. A 23-French nondeflectable sheath (Micra™; Medtronic, Minneapolis, MN, USA) was advanced into the right atrium, through which a 12-French deflectable sheath (FlexCath Advance™; Medtronic) was placed transseptally. A 2.4 mm × 20 cm Raptor™ grasping device (US Endoscopy, Mentor, OH, USA) **(Figure 1)** was advanced through the 12-French sheath to grasp the WATCHMAN™ device. With sustained traction, the device was dislodged from the left atrial appendage into the 23-French sheath **(Figure 1 and Video 3)**. This older WATCHMAN™ device was negative for any surface thrombi or debris. Subsequently, placement of a new 24-mm WATCHMAN™ device was performed with good apposition; the temporary WATCHMAN™ device in the aorta was removed at this point. The procedural steps are summarized in **Table 1**. Follow-up TEE performed at four months confirmed stable device positioning without significant peridevice leakage.

In unusual instances, a left atrial appendage closure device may be found to have chronically migrated to an unfavorable position, requiring removal. This case demonstrates the safety and feasibility of WATCHMAN™ extraction using an endoscopic grasping tool and subsequent device re-implantation.^2,3^ We preferred the use of the Raptor tool given the chronicity of the previously placed WATCHMAN™ device, which may not have been suitable for retrieval using a snare or biopsy bioptome. The decision to place a WATCHMAN™ device temporarily in the ascending aorta was driven by the limitations of currently available embolic protection devices to prevent peripheral embolization.

## Figures and Tables

**Video 1:** **A–C**: Transesophageal echocardiography shows the malpositioned WATCHMAN™ device.

**Video 2:** **A and B**: A WATCHMAN™ device was positioned temporarily in the ascending aorta for embolic protection.

**Video 3:** The malpositioned WATCHMAN™ device is removed with the grasping tool under fluoroscopy.

**Table 1:** Description of the Step-by-step Approach Adopted for WATCHMAN™ Extraction

Procedural Step	Purpose
TEE	Performed as the initial step to exclude thrombus in the LAA and LAA prior to pursuing further procedural steps
Vascular access	Placement of 8.5-Fr sheath in the left femoral vein, 11-Fr sheath in the left femoral vein, and 5-Fr arterial sheath with subsequent upgrade to 8-Fr in the left common femoral artery
Further exchange	The 8.5-Fr sheath was exchanged for an SL-1 sheath
Transseptal access	Using a Brockenbrough needle, the SL-1 sheath was exchanged for a 23-Fr Micra™ leadless pacemaker sheath; then, another FlexCath sheath was advanced via the leadless sheath into the LA
Arterial exchange	The 8-Fr arterial sheath was exchanged for the 14-Fr WATCHMAN™ sheath, which was placed in the ascending aorta
Embolic protection	A 27-mm WATCHMAN™ device was placed in the ascending aorta
WATCHMAN™ extraction	The Raptor™ device was then advanced via the 12-Fr Flex sheath to placement in a coaxial manner to grasp the older WATCHMAN™ device from the LAA
Sheath exchange	A new 14-Fr WATCHMAN™ sheath was then placed into the LA
TEE and LAA angiography	LAA measurements were collected and an angiogram of the LAA was created using a pigtail catheter
New WATCHMAN™ device implantation	A new 24-mm WATCHMAN™ device was delivered and positioned in the LAA; its positioning was further verified using TEE and fluoroscopy
Vascular access closure	The venous access site was closed using a figure-of-eight suture, while the arterial access route was closed using a Perclose ProGlide™ tool (Abbott, Chicago, IL, USA)

LA: left atrium; LAA: left atrial appendage; TEE: transesophageal echocardiography.
